# Haptoglobin related protein without signal peptide induces a new syndrome of renal salt wasting in Alzheimer’s disease

**DOI:** 10.1002/alz.089602

**Published:** 2025-01-09

**Authors:** John K Maesaka, Louis J Imbriano, Grant Candace, Nobuyuki Miyawaki

**Affiliations:** ^1^ NYU Long Island School of Medicine, Mineola, NY USA

## Abstract

**Background:**

A new pathophysiologic approach to evaluating hyponatremic patients identified the different causes of hyponatremia, including non‐hyponatremic patients with renal salt wasting (RSW). RSW was considered a rare to nonexistent syndrome until we found 24 (38%) of 62 hyponatremic patients in a general medical ward to have RSW. We induced RSW in rats by injecting plasma from 18 AD patients suspected to have RSW. We utilized lithium as a marker of sodium transport in the proximal tubule. All 18 AD patients below a mini‐mental state examination (MMSE) score of 12 had increasing excretion of lithium from 33 to 58%.

**Methods:**

The same renal clearance studies demonstrated natriuretic activity (NA) in AD serum. We subjected the serum with NA and normal control serum to proteomic analyses to identify the protein content in both sera.

**Results:**

Fractional excretion (FE) of sodium and lithium increased from 0.2% to 0.69 % and 24.4 to 52.5% in the control and AD sera, respectively. FElithium progressively increased from 33% to as high as 58% as MMSE decreased from 12 to zero. Phenotype specific haptoglobin 1‐1, and 2‐2, kininogen, thrombospondin, PROZ, alpha‐1 microglobulin/bikunin, and retinol binding protein did not have NA. Haptoglobin related protein with signal peptide did not have NA but there was a robust dose response to haptoglobin related protein without signal peptide (HPRWSP). Am Jour Med Sci 2021;361:261‐268

**Conclusion:**

It appears that every patient below an MMSE of 12 becomes progressively dehydrated. Studies are underway to determine whether HPRWSP can serve as a biomarker of RSW in Alzheimer’s disease and to determine the prevalence of RSW at different stages of dementia.

